# Sesquinary catenae on the Martian satellite Phobos from reaccretion of escaping ejecta

**DOI:** 10.1038/ncomms12591

**Published:** 2016-08-30

**Authors:** M. Nayak, E. Asphaug

**Affiliations:** 1 Red Sky Research, LLC, 67 Northland Meadows Drive, Edgewood, New Mexico 87015, USA; 2 Department of Earth & Planetary Sciences, University of California at Santa Cruz, 1156 High Street, Santa Cruz, California 95064, USA; 3 School of Earth & Space Exploration, Arizona State University, 781 S Terrace Road, Tempe, Arizona 85281, USA

## Abstract

The Martian satellite Phobos is criss-crossed by linear grooves and crater chains whose origin is unexplained. Anomalous grooves are relatively young, and crosscut tidally predicted stress fields as Phobos spirals towards Mars. Here we report strong correspondence between these anomalous features and reaccretion patterns of sesquinary ejecta from impacts on Phobos. Escaping ejecta persistently imprint Phobos with linear, low-velocity crater chains (catenae) that match the geometry and morphology of prominent features that do not fit the tidal model. We prove that these cannot be older than Phobos' current orbit inside Mars' Roche limit. Distinctive reimpact patterns allow sesquinary craters to be traced back to their source, for the first time across any planetary body, creating a novel way to probe planetary surface characteristics. For example, we show that catena-producing craters likely formed in the gravity regime, providing constraints on the ejecta velocity field and knowledge of source crater material properties.

## Abstract (web summary)

The origin of the numerous linear grooves and craters that litter the Martian moon Phobos' surface remains enigmatic. Here, by modelling low-velocity escaping ejecta from impacts to Phobos, the authors show that several of these chains can be explained by reimpacting sesquinary ejecta shortly after ejection.

## Graphical abstract

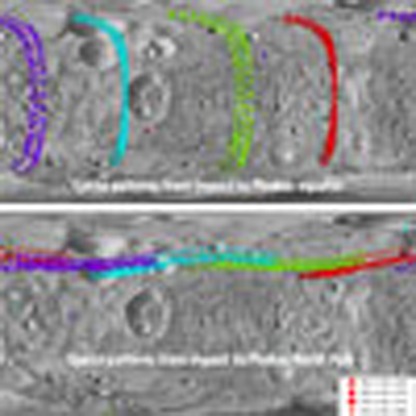

Ejecta escaping from an impact on a natural satellite often goes into orbit about the primary and can reimpact the satellite or its companions after an extended period. These ‘sesquinary' impacts^1^ are slower than primary impacts, but faster than the satellite escape velocity. Because they spend time in orbit they do not simply radiate from their primary crater as secondary craters do; nonetheless, their close dynamical association with the satellite can give them a unique geometrical distribution. Like secondary craters, sesquinaries are probes of the primary ejection process, but are also bound to the dynamics of the planet-satellite system. Unlike secondaries, to date no sesquinary crater has been traced back to its primary.

Phobos, the 26 × 22 × 18 km battered moon of Mars, is covered in parallel linear features whose orientation is, for the most part, aligned with de-orbiting tidal stresses as Phobos spirals closer to Mars. As the tidal bulge grows, surface stresses increase and cause striations^2^,^3^. However, many of Phobos' linear features do not align with any interpretation of tidal stress, giving rise to alternative models such as impact fractures related to the formation of Stickney^4^, the largest crater on Phobos, ejecta from Mars basin formation^5^, pitted regolith from bouncing boulders^6^ and drainages opening up into a fractured substrate^7^. Even with recent improvements incorporating two-layer tidal stresses from orbital decay^3^, a subset of prominent hemispherical crater chains crosscut predicted stress fields and bear a closer resemblance to distal secondary crater chains on the Moon, except there is no apparent source crater. They also resemble the tidal catenae on Ganymede and Callisto that are the result of cometary disruption inside the Roche limit of Jupiter^8^. We propose these features on Phobos are a novel kind of structure intermediate between these two phenomena, which we call ‘sesquinary catenae'.

We classify four kinds of satellite impacts. A primary impact is by a bolide from outside the planet's sphere of influence; these are generally the fastest. A secondary impact is by a fragment ejected from a primary crater; these are the slowest cratering events, no faster than a fraction of the satellite's escape velocity *v*_esc_. Such craters radiate from the primary and form linear chains and clumps. A sesquinary impact is formed by crater ejecta that escapes but remains in orbit in the system; its impact velocity is intermediate between *v*_esc_ and the orbital velocity *v*_orb_. When the satellite is far from the planet, sesquinaries can produce primary-like crater morphology. Intermediate between sesquinary and secondary is the so-called dosquinary, where the ejecta reimpacts after spending only a few orbits aloft. These can be thought of as the slowest possible sesquinaries, but not quite secondaries, since the gravitational influence is primarily that of the planet. These are limited to satellites that orbit close to the planet.

Sesquinaries/dosquinaries from Phobos are especially interesting for three reasons: first, the escape velocity *v*_esc_ is of order ∼10 m s^−1^, hundreds of times slower than its orbital velocity *v*_orb_ ∼2.2 km s^−1^. The slowest escaping ejecta, which comprise the major mass fraction, cannot stray far from the satellite. Second, the escaping ejecta are subject to powerful orbital and tidal distortion, since the current semimajor axis *a*∼2.77*R*_Mars_ is significantly inside the classical Roche limit *R*_roche_∼3.19*R*_Mars_ (ref. 9). Third, as we shall show, the timescale for reaccretion is so short that ejected particles can be reaccreted before they have time to disperse. This leads to the curious geomorphic phenomenon of sesquinary catenae, each linked to a particular source crater on Phobos, and sets these features apart from crater chains on bodies beyond deep planetary gravity wells of planets, such as Eros^10^, Gaspra and Steins^11^. Analytical formulations have been used to study the dynamics of escaping and reaccreting ejecta^12^,^13^,^14^, but to precisely predict sesquinary reimpact locations we require an ephemerides formulation. We consider multiple orbital configurations around Mars to evaluate variations in Mars-Phobos orbital geometry and inferior or superior conjunctions with Deimos at the time of the primary ejecta-producing impact. Since the significant percentage of mass escaping Phobos is ejected slower than 100 m s^−1^ (92%, Methods section), we track in precise detail the component ejected with velocities from *v*_esc_∼11 to 100 m s^−1^. Planetocentric latitude and longitude of each impact location is extracted from the evolution model.

## Results

### Primordial versus modern orbit

**Figure 1:**
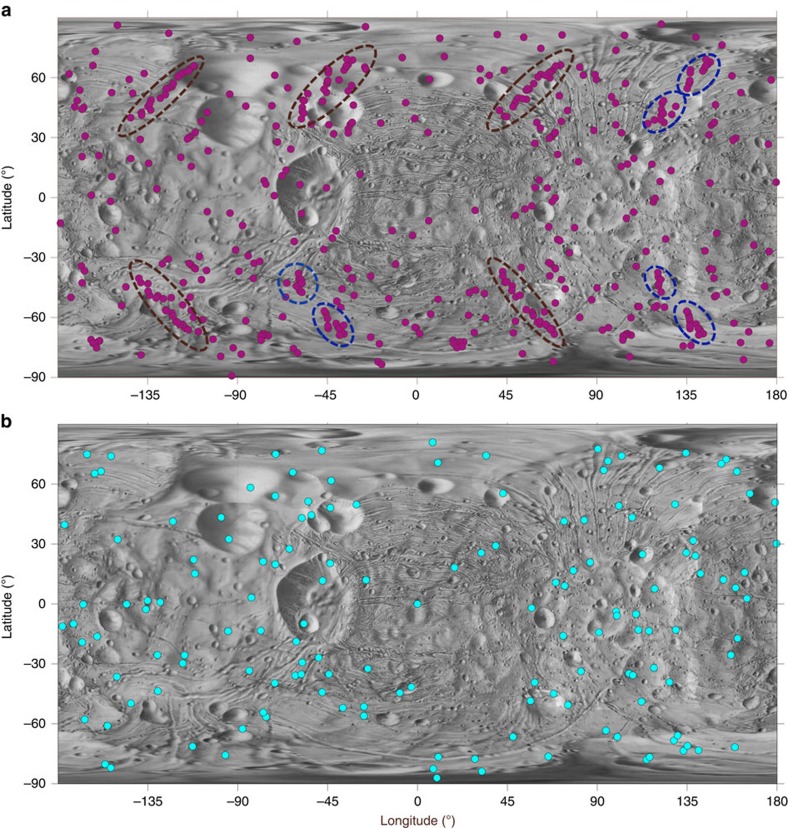
Reaccretion map for the Stickney impact in Phobos modern and primordial orbits. (**a**) Ejecta from Phobos in its modern orbit, *a* ∼2.77*R*_Mars_; multiple linear strings of reimpacts are noted in both hemispheres (brown and blue ellipses). (**b**) Ejecta from Phobos in its primordial orbit near the Mars synchronous line, *a* ∼6*R*_Mars_; similar reaccretion patterns are not seen. All ejection velocities range from 11 to 100 m s^−1^, azimuth ***β***∈ [0:*π*/6:2*π*].

We find that a majority of the mass ejected from Phobos at low velocity (<100 m s^−1^) reaccretes; the rest impacts Mars or leaves the system. A minor fraction (<1%) impacts Deimos at randomized locations. We characterize Stickney, the largest crater on Phobos, which either formed geologically recently or when Phobos was in a more distant orbit close to the synchronous line^15^,^16^,^17^. Figure 1a shows the reimpact map for Stickney had it occurred in Phobos' present orbit, with a mean reaccretion time of ∼22 h, where *a*∼2.77*R*_Mars_. Catenae from Stickney are clearly distinguishable. Figure 1b shows the reimpact map for a primordial *a*=6*R*_Mars_ (ref. 15). Here, with a mean reaccretion time of ∼32 years and max of 88 years, individual reaccreted particles have lost their geometrical association due to the much longer flight time. A study of sesquinaries originating from Deimos finds that ejecta particles reaccrete over ∼500 years and do not form crater chains^18^. Therefore, for Phobos' modern orbit inside the Roche limit, reimpacts are not only more frequent than in a primordial orbit but also occur over shorter timescales (days) and are recognizably coherent, forming chains.

### Nature of reimpacting ejecta

**Figure 2:**
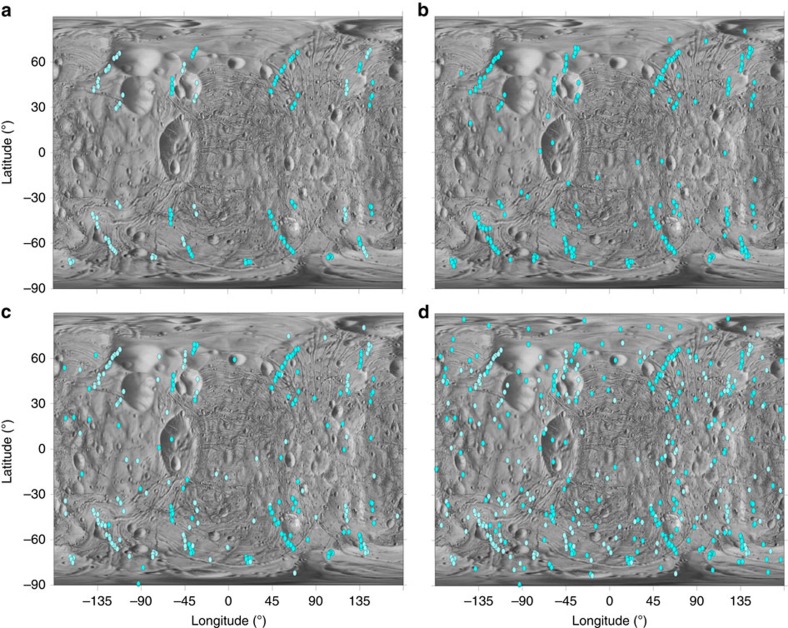
The evolution of catenae with increasing velocity of ejection from Phobos. Maps show reimpact locations for sesquinary impactors with ejection velocity greater than Phobos escape velocity and lesser than (**a**) 20, (**b**) 25, (**c**) 30 and (**d**) 100 m s^−1^. Catenae-like formations are evident from particles ejected at lower velocities. As ejection velocity increases past 30 m s^−1^, reimpacts become less correlated to catenae-like structures. The source of the catenae is therefore very low-velocity particles. The primary impact location is Stickney and azimuth ***β***∈ [0: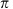/6:2*π*].

**Figure 3:**
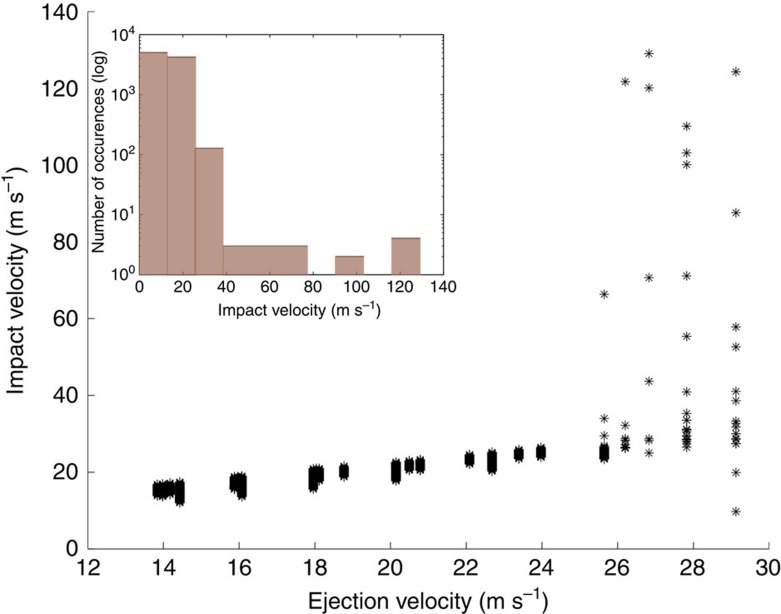
Impact versus ejection velocity for sesquinary impactors. Ejecta correspond to simulations in all panels of Fig. 5. The majority of impacts occur at a velocity comparable to their ejection velocity, that is, between 11 and 30 m s^−1^. However, a small fraction experience acceleration due to greater interactions with Mars' gravity (inset); these have a correspondingly longer accretion time. On the basis of these results, sesquinary impact craters are largely expected to have an appearance similar to secondary craters noted in image surveys of Phobos.

The pattern of reimpacts as pictured against increasing ejection velocity (Fig. 2) shows that the catenae originate from very-low-velocity particles just above the escape velocity of Phobos. At ejection velocities of <25 m s^−1^, nearly all reimpacts cluster in catenae-like patterns; above 30 m s^−1^, a few times *v*_esc_, reimpacts are less correlated to catenae-like structures. Studying the reimpact velocities (Fig. 3), we find that they are correspondingly higher than *v*_esc_ (refs 19, 20) but sufficiently low that craters produced are expected to morphologically resemble secondary craters. In addition, though the mean reaccretion time for all Phobos-impacting particles is ∼22 h, the large majority of catena-forming impacts occur in ∼6–14 h.

As such, these are a special case of sesquinary impacts (dosquinaries). Although the impactors temporarily escape the gravitational influence of Phobos, they exhibit behaviour that may be best described as intermediate between a secondary and traditional sesquinary impactor. Since 80% of the total mass that escapes Phobos during an impact is ejected at speeds of <30 m s^−1^ (Methods section), this mass influx is a mechanism capable of significant impact to the surface geology; for detailed characterization of catena production we now focus on 10–30 m s^−1^ streamlines.

Next, we attempt to establish if the catenae are a frequent phenomenon by searching for a lower limit of impact that results in the creation of catenae-like structures. We generate <30 m s^−1^ reimpact maps for ejecta from primary craters of 1, 3 and 5 km in diameter (Stickney: ∼10 km diameter). As seen in Supplementary Fig. 1, catenae are predicted to form as a byproduct of sesquinaries from primary craters at least as small as 1 km in diameter, and likely smaller. The saturated (at least down to 300 m craters^21^) surface of Phobos suggests that the creation of low-velocity, clustered linear impact structures from sesquinary ejecta is a relatively frequent process; catena formation is approximately as frequent as gravity-regime crater formation, where most of the escaping ejecta mass is just barely escaping (<30 m s^−1^). This suggests that linear chains of low-velocity impact structures are a relatively frequent process on modern Phobos, each correlated with a source crater.

**Figure 4:**
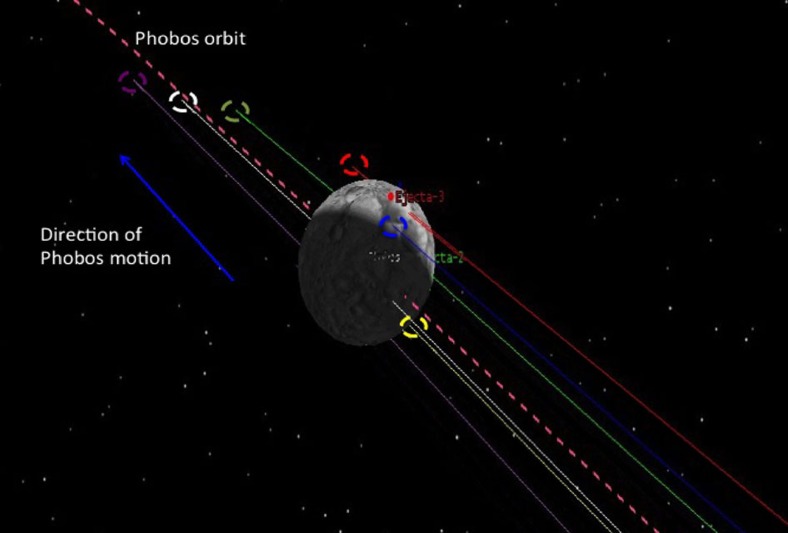
Visualization of the orbital history of randomly selected particles. Shown are the trajectories of randomly selected projectiles ejected at 11–30 m s^−1^, which impact over a relatively short time in a grouped fashion. As Phobos moves along its orbit (towards the top of the figure, dashed pink line), ejected particles in the vicinity of Phobos' orbit (purple, white, green, red and yellow lines) are reaccreted to the satellite. Dashed circles represent planetocentric impact points, illustrating how hemispherical catena (Fig. 5) may be formed.

It is interesting to note that a kilometre-sized crater in the strength regime would create faster ejecta, consequently producing less-reaccreting ejecta^22^ and few (if any) crater chains. This analysis therefore allows us to probe the dynamics of crater ejecta in a way not done before, for instance proving that catena-producing craters formed in the gravity regime (in deep regolith) and are likely not much older than Phobos' current orbit (<50 Ma). Figure 4 illustrates how orbital ejecta lingering in the vicinity of the Phobos orbit can be swallowed up in hemispherical patterns that lead to chain or catena-like reaccretion patterns (for example, Fig. 1). Tracing the precise orbital history of multiple particles shows that ejected particles transition from the gravitational influence of Phobos to Mars for a period of between 1 and 5 orbits before subsequent impact, reaccreting before they have time to disperse. The mean reaccretion time of ∼22 h is ∼3 times the orbital period of Phobos; for <100 m s^−1^ impact velocity events, the mean reaccretion time is ∼6–14 h, or ∼1–2 times the orbital period of Phobos.

The higher the ejection velocity (Fig. 3) the higher the corresponding scatter in the locations of reaccretion (Fig. 2). This correlates to longer flight times and greater interactions with Mars-dominated gravity, as opposed to the low-velocity impactors, which escape Phobos-centred gravity but do not stray far from the orbit of the satellite before reimpact. Low-velocity impactors creating the catena appear to be dosquinary in nature, that is, intermediate between typical secondary and sesquinary impactor behaviour.

**Figure 5:**
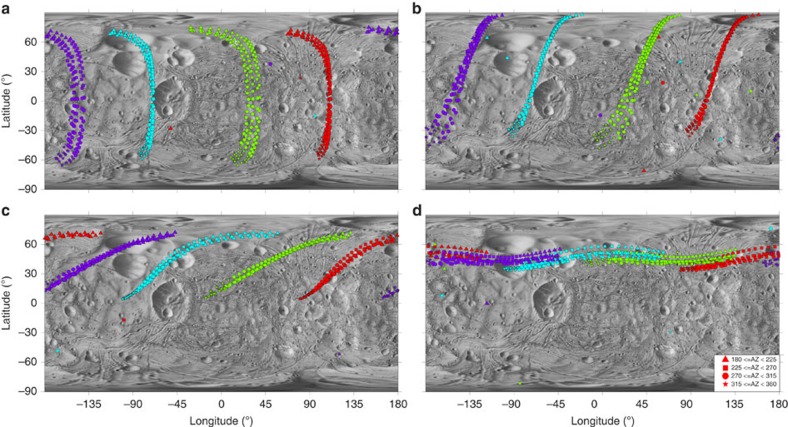
Catenae orientations change from vertical to horizontal depending on the latitude of the primary impact. Maps show resultant catenae from primary impacts on Phobos at the prime meridian and (**a**) 0° N, (**b**) 30° N, (**c**) 60° N and (**d**) 85° N. Resulting orientations are the mirror inverse of those from impacts to the Southern Hemisphere (compare with Supplementary Fig. 5). Variations with orbital geometry at primary impact can be seen, namely, when Phobos is at Mars periapsis (red), apoapsis (blue), halfway between periapsis and apoapsis along the ascending node (purple) and descending node (green). Ejection velocities are 11–30 m s^−1^; the source crater is 3 km in diameter. Shapes denote ejection azimuth (legend); azimuths ***β***∈ [*π*: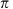/36:2*π*] are shown (compare with Supplementary Fig. 4).

Given a low-velocity ejection bracket (11–30 m s^−1^, Fig. 5), we find that the azimuth of ejection controls the length and coherence of the catenae. From the ejection azimuths for reaccreting particles, it can be seen that different azimuths result in different ‘sections' of the catena being formed (shape legend, Fig. 5). The formation of an axisymmetric cone, with ejecta along every outbound azimuth, is of course an idealized case. In reality, depending on the geometry of the primary crater formation (and resulting streamlines), catenae may range from highly focused to ‘patchy'. If no particle is ejected between the azimuths of (say) 30° and 45°, there will be a corresponding ‘gap' in the catena. The location of this gap can be inferred from the respective shape legends. Inversely, given a suspected catena on Phobos, this makes it possible to infer the properties of the primary impact.

### Grid search varying the primary crater location

Good correlation exists between models of tidal stresses induced by the orbital decay of Phobos towards Mars and several groove families, but cannot match a number of grooves across the surface^3^. Can the observed catenae detailed in this work account for these misfit grooves? Even among this subset of grooves, a wide variety of orientations are seen^23^. To determine if sesquinary catenae can be responsible for all these varied orientations, we investigate the effect of ejecta originating from sources other than Stickney.

We create an equidistant longitudinal and latitudinal grid, ranging from 90° N to 90° S in 30° increments and 180° W to 180° E in 90° increments, for a total of 35 grid points. This span accounts for variations between the leading and trailing apex of Phobos, as well as the sub- and anti-Mars points. Since it has already been shown that the size of the origin crater has no effect on the geometry of the resulting reaccretions, we choose the velocity distribution from a 3 km diameter crater as the standard distribution and ‘release' it from each grid point.

Our results show that the longitude of the primary crater has no effect on reaccreting catenae for impacts in both the Southern Hemisphere (Supplementary Fig. 2) and Northern Hemisphere (Supplementary Fig. 3). In these figures, note that we have restricted ourselves to showing results for *β|β*∈ [*π:π*/36:2*π*) for clarity; results are mirrored for *β|β*∈ [0:*π*/36:*π*). This can be seen by comparing the panels of Supplementary Fig. 5, the reimpact map for the Stickney impact.

While longitude of the primary crater has little effect, the latitudinal location of the primary impact has a direct relation to the orientation of the catenae. Figure 5 shows catenae resulting from primary impacts at 0°, 30°, 60° and 85° N latitude. An interesting pattern emerges: polar primary impacts create horizontal catenae, while vertical chains result from equatorial primary impacts. These results are mirrored in the Southern Hemisphere (Supplementary Fig. 5). The range of possible catenae orientations, from horizontal to vertical, show that low-velocity sesquinary impactors could indeed match the orientation of those grooves that do not fit a tidal evolution origin. Orientations are mirrored across primary impacts to either hemisphere, which can be seen by comparing Fig. 5 (Northern Hemisphere) to Supplementary Fig. 5 (Southern Hemisphere). From the variation in orbital geometries at the time of the primary impact, the location of the catenae can shift longitudinally, depending on the location of the primary impact along Phobos's orbit around Mars.

### Tracing catenae back to a source crater

**Figure 6:**
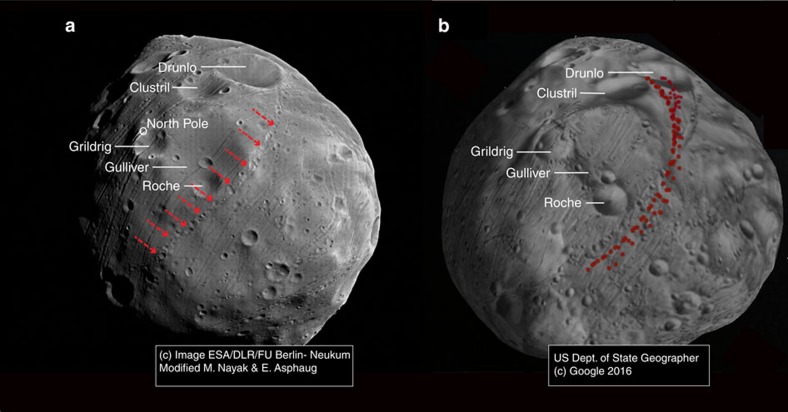
Tracing an observed catena back to its source crater. (**a**) Spacecraft image of Phobos (photo credit: ESA/Mars Express) showing the observed catena of interest (red arrows); (**b**) reimpact map for a primary impact at Grildrig, azimuth ***β***∈ [0: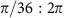) rendered in three dimensions. Relative sizes and orientations between **a** and **b** are similar and may be correlated from Drunlo, Clustril, Grildrig, Gulliver and Roche craters, respectively. From the correlation, the highlighted catena likely originates from sesquinary ejecta from Grildrig.

On the basis of the relationships of reaccreting catenae to a primary crater location, we can now match a catena to its source crater, not previously done for any planetary body. In doing so we can constrain the ejecta velocity field and provide knowledge of the material properties in the region of the source crater. For our case study, we choose one of the more prominent Phobos catenae, shown in Fig. 6a. Previously studied in the literature^21^ and hemispheric in extent, it is not obviously correlated with Stickney or any other crater, is morphologically similar to cratering expected for ejecta colliding at just above ∼*v*_esc_ and crosscuts the predicted stress field for tidal grooves^3^. This makes it a suitable test of the hypothesis that sesquinary catenae can match those grooves that do not fit tidal models.

In comparison with Fig. 5d, the highlighted catena is similar to reimpacts predicted for a near-polar primary source crater, narrowing the grid search to above 60° N. We model sesquinary ejecta from an impact at the 2.6 km diameter crater Grildrig (81° N and 196° W) and find a very close match to the observed catena (Fig. 6). This test case shows that reimpacting slow co-orbital ejecta can explain previously mysterious features not well explained by any previously proposed mechanism. These ejecta would be proximal to the crater on a ‘normal-gravity' body like the Moon, but on Phobos, they get pulled apart into strands before re-impact a matter of orbits later.

## Discussion

Our results support Grildrig's formation in the gravity regime and in deep regolith. Grildrig does show raised rims corresponding to gravity-regime production, is reasonably fresh, and of suitable diameter to produce the observed catena. When loose material is ejected at velocities just exceeding escape velocity, self-gravity of the material becomes a factor and leads to clump formation, as seen from asteroid family and binary formation simulations^24^,^25^. This is how an impact to deep regolith can release large ejecta fragments, which would then become sesquinary impactors. This is also why the catenae do not have cleanly discriminated crater forms, but rather clumpy streamers of interconnected features.

The rim of Grildrig shows craters similar in size to the catena in its vicinity; it is likely that reimpacting ejecta may have formed some of these. Better image data from a dedicated Phobos mission can enable studies of chronology in the relative sense, that is, evaluate the hypothesis that craters on the rim of Grildrig are coeval with catena craters associated with sesquinary ejecta from Grildrig. Certainly, if sesquinary ejecta is indeed the source of the indicated crater chain, there is little one can say about crater ages on Phobos from the consideration of sub-kilometre scale crater densities; age estimates from crater counting were previously used^26^ as an argument against a tidal origin^2^ for grooves.

In conclusion, catenae on Phobos created by low-velocity sesquinary impactors are persistent across the range of longitudinal, latitudinal, orbital and conjunctive variations, with distinctive resulting geometries. This in turn implies that catena formation is approximately as frequent as gravity-regime crater formation, where most of the escaping ejecta mass is just barely escaping (<30 m s^−1^). For such events, the fate according to our model is for material to impact in a linear chain. On the basis of Fig. 3, we expect the resulting crater morphology to be similar to secondary craters in nature, consistent with secondary crater chains and catenae noted on the Moon. The direct association of sesquinary catenae with source craters is an important and new kind of planetary data set, leveraging a 1:1 correspondence to constrain the dynamical and geologic history of Phobos in a novel way. Without applying any morphological criterion, we can say if a catena-like feature on Phobos is a good candidate for a sesquinary origin by asking if it follows the distinct trending direction that it must follow if it were sesquinary (Fig. 5). Based solely on an inspection of Fig. 5, initial guesses may be made at the latitude of that feature's possible source crater; a grid search similar to that performed for Grildrig would then narrow possibilities down to one source crater.

Significant craters without corresponding catenae might need to have formed when Phobos was in a more distant orbit, or in strength-controlled impacts with faster ejecta. Conversely, catenae without corresponding source craters may have formed when Phobos was in a different orbital or tidal-locking geometry with respect to Mars. The lack of correlation of major catenae with our predictions for Stickney (Fig. 1) suggest that Stickney formed when Phobos was more distant from Mars^27^. For example, long flight times for particles reaccreting to Deimos result in many reimpacts but no catenae on that body^18^. This does not preclude the possibility of impact craters on Phobos that are uncorrelated Stickney sesquinaries.

Discriminating sesquinary catenae from tidal stress-induced fissures^3^, or catenae-like features formed from regolith draining into fissures^7^, is the next step in Phobos surface science. For example, sesquinary catenae may exhibit raised rims typical of impact craters, whereas catenae-like features formed by regolith draining would not. Similarly, the walls of drainage pits would stand at the angle of repose, whereas sesquinary craters might be less steep. However, given the generally heterogeneous quality and resolution of Phobos images, and the lack of systematic mapping products (for example, ref. 28) applied to the Mars Express data sets^9^, discriminating catenae as sesquinary or otherwise solely on the basis of imaging is an expansive task, rendered further difficult by the fact that there may be multiple mechanisms for linear feature formation at play. There is a need for published topographic profiles of Phobos that reliably measure the slopes of Phobos features relative to the satellite's effective gravity (which can vary measurably across the surface of the moon), from which the distinct origin of a particular catena might be distinguished. The creation of these products is ongoing.

Using this, future work will start with systematic and objective geomorphic measurements of the directions, slopes, depths, and the sizes and spacings of linear pits and craters, to classify linear features on Phobos objectively (for example, into secondary ejecta-like streamers versus fissures and so on), analogous to Morrison *et al.*^28^ but applied to modern Phobos data^9^. These classified features will then be compared with model predictions, especially sesquinary catena predictions from fresh-looking source craters in present and geologically recent orbits, groove predictions from the tidal model^3^ and any other viable mechanisms. This study also has potential implications for other closely bound satellite systems, especially Pluto and Charon, whose tidal locking and close proximity leads to the possibility of similar short-timescale reaccretions collecting preferentially onto facing hemispheres.

## Methods

### Mars gravity system integrator

Ejecta are integrated in an ephemeris-centred frame for an arbitrarily chosen primary impact epoch, incorporating solar third body perturbations and a 20 × 20 Mars gravity harmonic model^29^. Our model accounts for changes in the rotation and longitude rate of Phobos, precession of the argument of periapsis and longitude of the ascending node, and its non-spherical shape (triaxial ellipsoid with semi-major axes of 13 × 11.4 × 9.1 km (ref. 30)). Integration time is capped at 10 years to permit use of a seventh-order Runge–Kutta integrator without excessive approximations to the perturbed Hamiltonian^31^.

### Ejecta velocity distribution

For the ejected material, we model streamlines ejected by a primary impact on Phobos using an axisymmetric *Z*-model formulation^32^,^33^. Though limited by its neglect of interactions across streamlines, the *Z*-model reasonably approximates several experimentally observed features^34^,^35^. The limitations of a *Z*-model implementation are discussed at length in ref. 34. Our application is only concerned with ejecta streamlines that escape Phobos, and is unaffected by the details of cratering flow beneath the ground plane, surface material mixing during ejection, or direct retention and emplacement of deposits^18^. Therefore the *Z*-model provides a suitable level of insight into an outbound velocity distribution; approximations made are unlikely to alter quantitative results. Hydrocode studies of the Stickney impact (∼10 km diameter) find *Z*=3.5 to suitably represent cratering flow^22^, adopted here. All streamlines are ejected at a constant angle of 56.3°, measured from the horizontal, set according to the relation^32^:


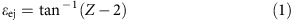


Outbound radial (*v*_*r*_) and vertical (*v*_*z*_) ejection velocities vary inversely with distance from the centre of the crater *r* as^32^


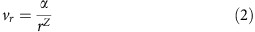



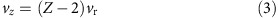


where


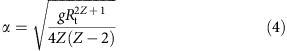


and *g* is the acceleration due to gravity on Phobos (0.0057, m s^−2^). The cratering flow is ultimately dependent on the transient crater radius, calculated as *R*_t_=0.65 *R*_f_, where *R*_f_ is the final radius of the crater^34^. For example, in Fig. 1, the Stickney primary impact is simulated using a final radius of 5.05 km (ref. 21) and a transient crater radius of 3.28 km. The number of streamlines *n* is chosen to yield a suitably dense distribution of velocities greater than the Phobos escape velocity. Setting *R*_min_=0 and varying *R*_min_≤*r*≤*R*_t_, *n* streamlines evenly spaced in radius are generated within a crater of choice. Converting streamlines to axisymmetric coordinates, radial and vertical coordinates are^34^


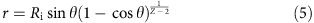



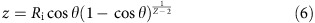


where *θ* is the angle from the vertical {*θ*|*θ*∈0:*π*/2} and


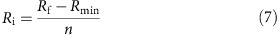


The *Z*-model yields a two-dimensional axisymmetrical velocity distribution; we rotate this around the azimuthal direction at 5°–30° azimuth (*β*) intervals to create a three-dimensional velocity distribution. This three-dimensional outbound velocity distribution is tied to the crater being studied, in the Topocentric Horizon frame, adapted from the South-East-Zenith frame^18^,^36^. This frame is centred at the crater, with the *x* axis pointing south and aligned with the meridian passing through the crater. The *x*–*y* plane is tangent to the surface and points along the local latitude circle. Completing the right-handed system, the *z* axis points radially outward from the impact site towards the ‘local' zenith^18^. For use with the Mars gravity system integrator, these coordinates are then rotated into the Mars Centered Inertial frame through the Phobos-centred Phobos-fixed and Phobos-centred inertial frames, respectively. Details of coordinate transformations and relationship between the frames may be found in appendix A of ref. 18. *ɛ*_ej_ is the elevation angle of the ejection velocity vector; from the *Z*-model *ɛ*_ej_=56.3°. *β*_ej_ is the azimuth of the ejection velocity vector and is measured from the North, positive clockwise as viewed from above the site such that 0≤*β*_ej_≤2*π*, and varies from *β|β*∈[0:*π*/6:2*π*) for a total of 13 possible azimuths to *β|β*∈[0:*π*/36:2*π*) for a total of 71 possible azimuths (see figure captions for specific details).

### Changing the *Z*-model ejection angle

Equation (1) fixes the ejection angle for the velocity distribution: we investigated whether this affects the outcome of our simulations. Supplementary Fig. 6 shows the reaccretion map for a simulation in which the ejection angle of the outbound ejecta is allowed to stochastically vary between *ɛ*_ej_=45° and 65°, that is, for *Z*-model numbers between 3≤*Z*≤4.14. This is overplotted on top of results for a simulation, which uses our standard values of *ɛ*_ej_=56.3° and *Z*=3.5. The similarity between the results shows that changing ejection angle or *Z*-number does not change our qualitative results; catena-like formations are still expected. However, the ejection angle may represent a constraint on the crater ejection mechanism; varying the *Z-*number may further improve the fit of our modelled catena to, for example, ejecta from the Grildrig crater.

### Mass–velocity distribution

For gravity-dominated cratering, the volume ejected faster than a given velocity is


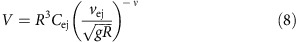


where *v*_ej_ is the ejection velocity and *R* is the final radius of the crater^28^,^37^. A sand-like surface is well represented^35^ by *ν*=1.2. Mass–velocity distributions from experimental impacts into granular targets^38^ find *C*_ej_=0.25, a value that correlates well to other published values^39^,^40^,^41^ between 0.25 and 0.36. We adopt *C*_ej_=0.3 and *ν*=1.2. Using equation (1) and a Phobos density of 1.9 g cc^−1^ (refs 42, 43), the mass ejected within each velocity bin is calculated. For the Stickney impact, of the 1.5 × 10^13^ kg ejected faster than the Phobos escape velocity, only 1.2 × 10^12^ kg (8%) is ejected faster than 100 m s^−1^. Similarly, with 2.72 × 10^12^ kg ejected faster than 30 m s^−1^, 80% of ejecta mass with sufficient velocity to escape Phobos leaves between 11.3 and 30 m s^−1^. These numbers are nearly identical for craters down to 1 km in diameter formed in the gravity regime.

### Data availability

Raw data for reaccretion maps and/or relevant computer code is available from the authors on request.

## Additional information

**How to cite this article:** Nayak, M. & Asphaug E. Sesquinary catenae on the Martian satellite Phobos from reaccretion of escaping ejecta. *Nat. Commun.* 7:12591 doi: 10.1038/ncomms12591 (2016).

## Supplementary Material

Supplementary Information Supplementary Figures 1-7
