# Supplementary material for: Sesquinary catenae on the Martian satellite Phobos from reaccretion of escaping ejecta
Source: Nat Commun. 2016 Aug 30;7:12591. doi: 10.1038/ncomms12591 (PMC5013556; doi:10.1038/ncomms12591)
Supplement: Supplementary Information — Supplementary Figures 1-7 [file ncomms12591-s1.pdf]

Supplementary Information: Sesquinary Catenae on the Martian Satellite Phobos from Reaccretion of Escaping Ejecta

M. Nayak and E. Asphaug

Supplementary Figures

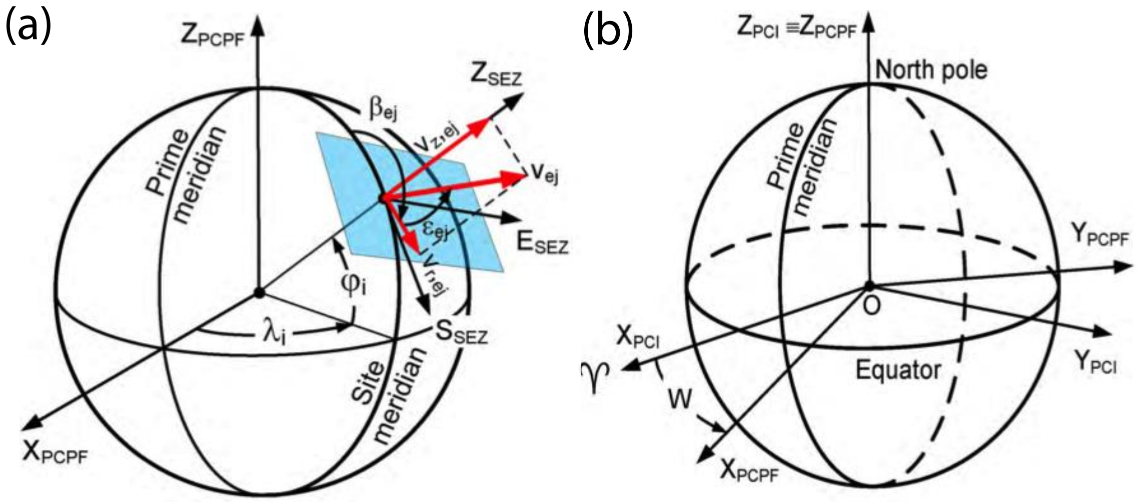

Supplementary Fig. 1. Relationship between reference frames. (a) Relationship between Phobos Centered Phobos Fixed (PCPF) frame and the South-East-Zenith (SEZ) frames; (b) Relationship between Phobos Centered Inertial (PCI) and PCPF frames. The fundamental plane is highlighted in blue.

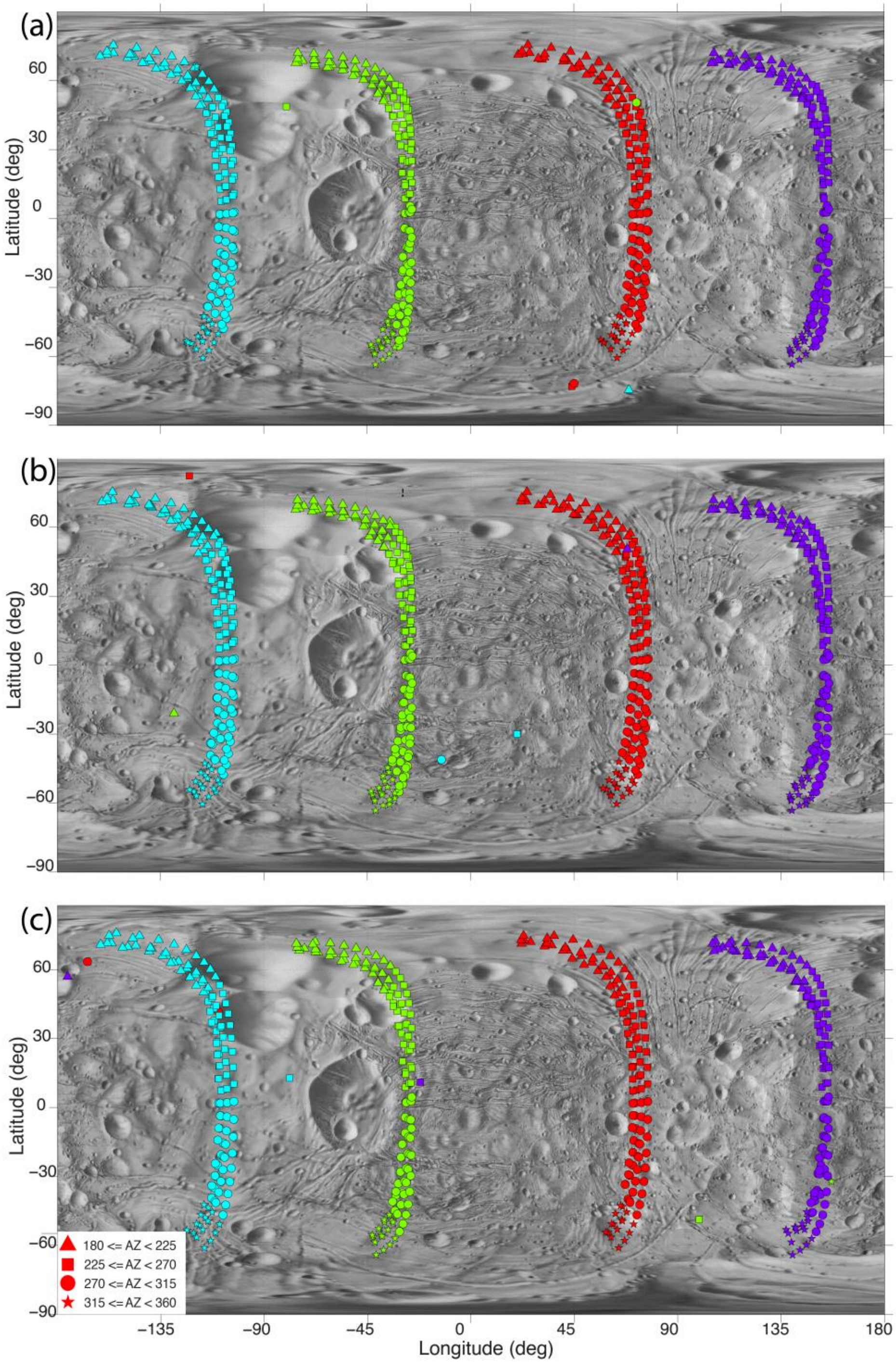

Supplementary Fig. 2. Impact of primary crater size on resultant catenae. Maps show resultant catenae from velocity distributions centered at the location of Stickney but with Z-model crater sizes of diameter (a) 5 km; (b) 3 km and (c) 1 km. Reimpacting particles have ejection velocities of 11-30 ms^{-1}; no change is evident between varying crater sizes in this velocity range. Craters as small as 1 km in diameter appear capable of creating catenae-like structures. Ejection azimuth β is henceforth restricted (compare to Supplementary Fig. 5) to $\beta \in [\pi: \pi/36: 2\pi)$; shapes denote ejection azimuth of reaccreted particles (legend, lower left). Changes with orbital geometry configurations at

the time of the primary impact are shown, namely, when Phobos is at Mars periapsis (red), apoapsis (blue), halfway between periapsis and apoapsis along the ascending node (purple) and the descending node (green).

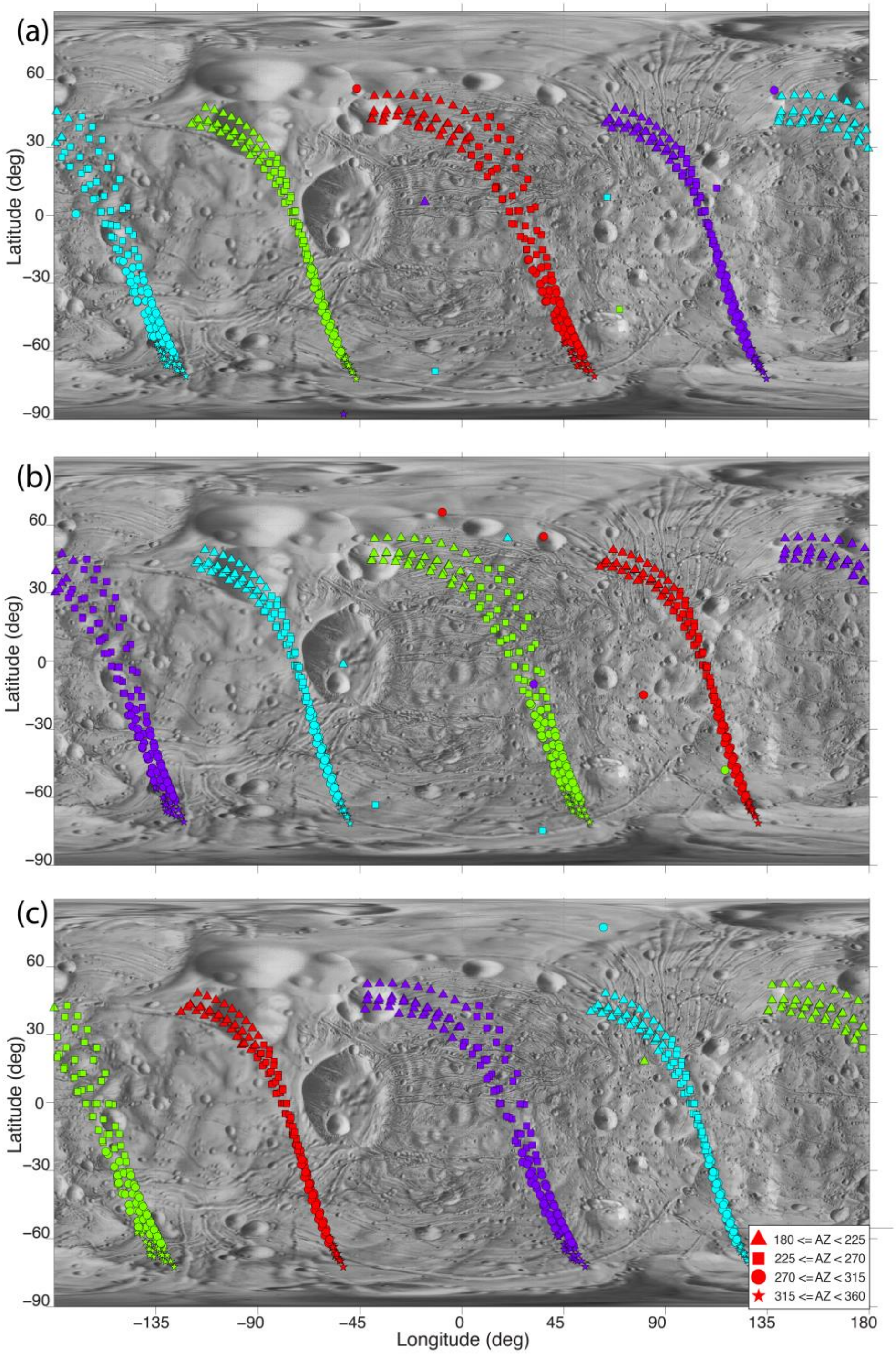

Supplementary Fig. 3. Impact of primary crater longitude (southern hemisphere) on resultant catenae. Maps show resultant catenae for a primary impact at latitude 30° S; longitude ranges between (a) 90° W; (b) prime meridian; (c) 180° E. Large changes in primary impact crater longitude have no effect on the orientation of the resulting catenae

and are degenerate with orbital phasing of Phobos around Mars. Reimpacting particles have ejection velocities of 11-30 ms^{-1} and $\beta \in [\pi: \pi/36: 2\pi)$. Colors and shapes denoting the ejection azimuth are as in Supplementary Fig. 2.

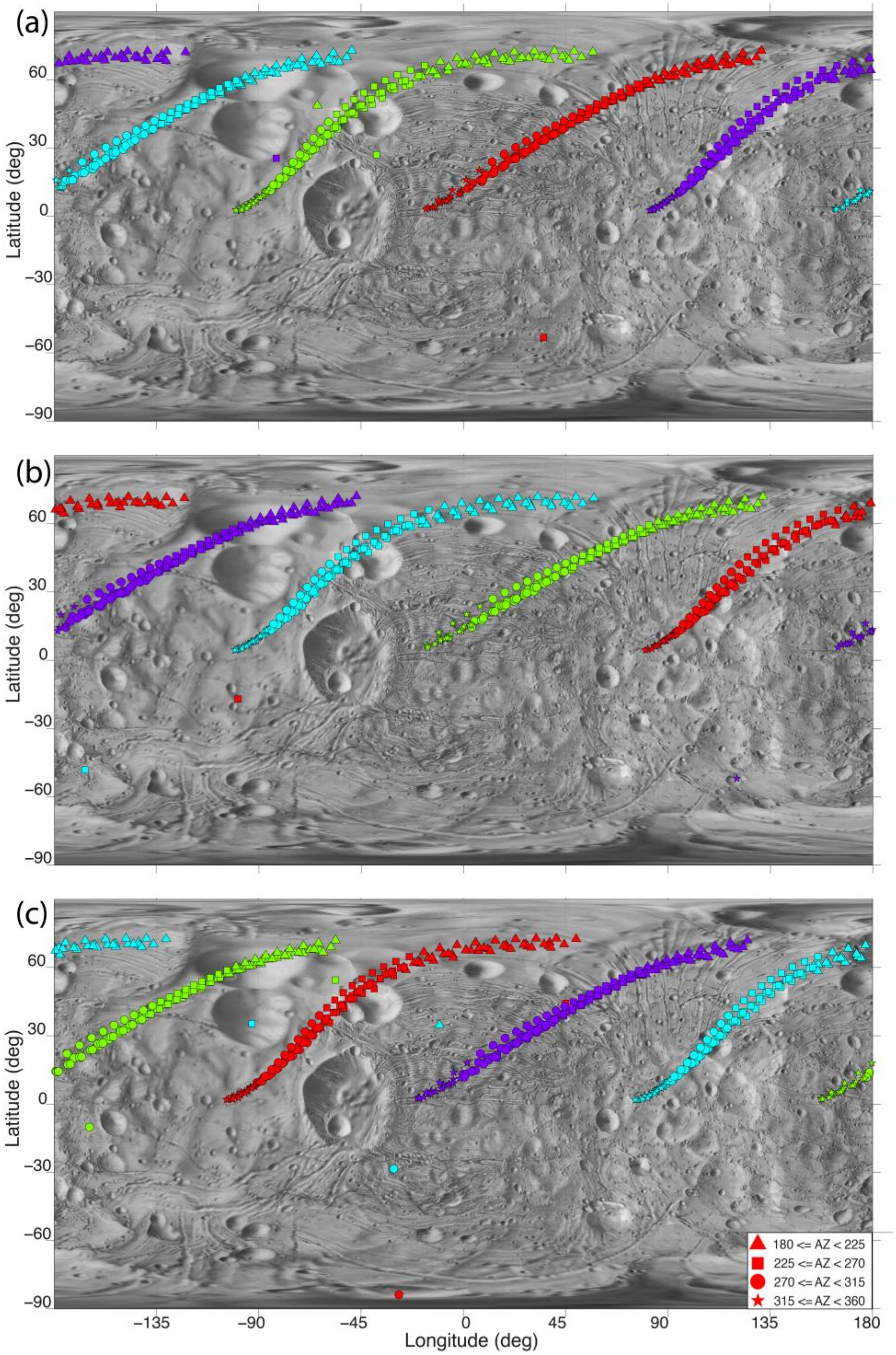

Supplementary Fig. 4. Impact of primary crater longitude (northern hemisphere) on resultant catenae. Maps show resultant catenae for a primary impact at latitude 60° N; longitude ranges between (a) 90° W; (b) prime meridian; (c) 180° E. Large changes in primary impact crater longitude have no effect on the orientation of the resulting catenae

and are degenerate with orbital phasing of Phobos around Mars. Reimpacting particles have ejection velocities of 11-30 ms^{-1} and $\beta \in [\pi\colon \pi/36\colon 2\pi)$. Colors and shapes denoting the ejection azimuth are as in Supplementary Fig. 2.

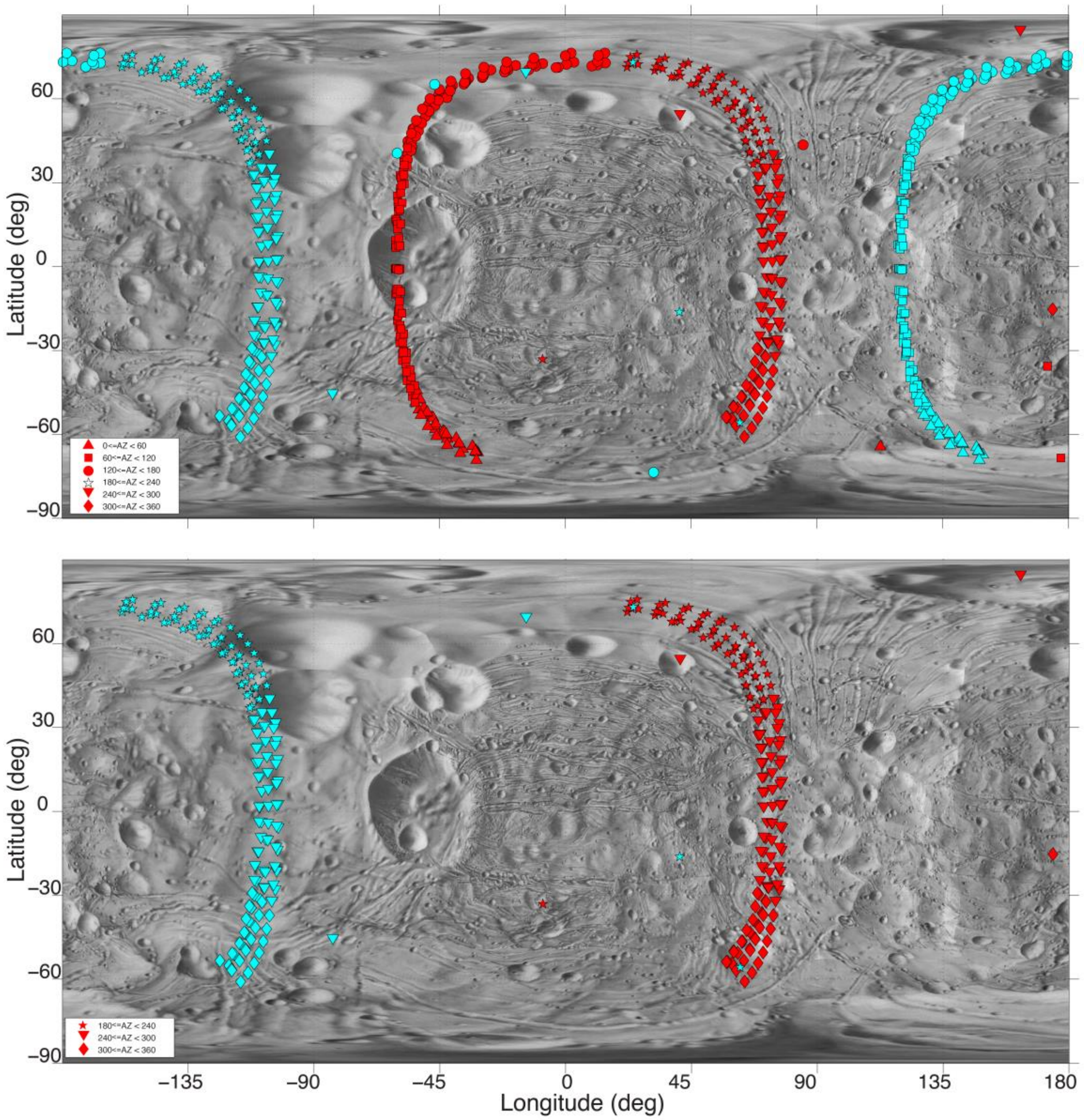

Supplementary Fig. 5. Comparing resultant catenae with azimuths of ejecta release. Maps show resultant catenae from the Stickney impact (centered at 1° N, 46° W) from particles with ejection velocities from 11-30 ms^{-1}. Colors are as in Supplementary Fig. 2. (**Top**) Azimuths of ejected particles are $\beta \mid \beta \in [0\colon \pi/36\colon 2\pi)$; (**bottom**) compares catenae for azimuths restricted to $\beta \mid \beta \in [\pi\colon \pi/36\colon 2\pi)$. Shapes denoting ejection azimuth are in legends (bottom left) of both panels. Mirroring the catenae in the bottom panel yields the full picture; for clarity in comparing geometry configurations (GCs) we show the subset of azimuths in the bottom subfigure.

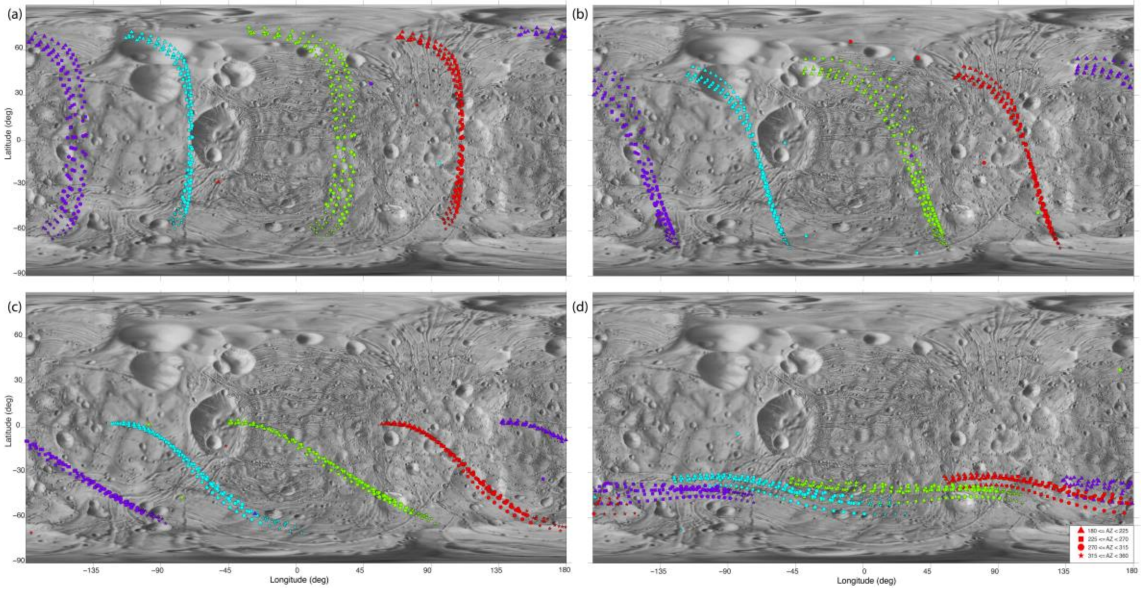

Supplementary Fig. 6. Impact of primary crater latitude (southern hemisphere) on resultant catenae. Maps show resultant catenae from primary impacts on Phobos at the prime meridian and (a) 0° S; (b) 30° S; (c) 60° S; (d) 85° S. Catenae orientation can change from near-vertical to horizontal, depending on the latitude of the primary impact, and mirror northern hemisphere impacts (compare to Fig 5, main text). Reimpacting particles have ejection velocities of 11-30 ms^{-1}, $\beta \in [\pi: \pi/36: 2\pi)$; colors and azimuth legend as in Supplementary Fig. 2.

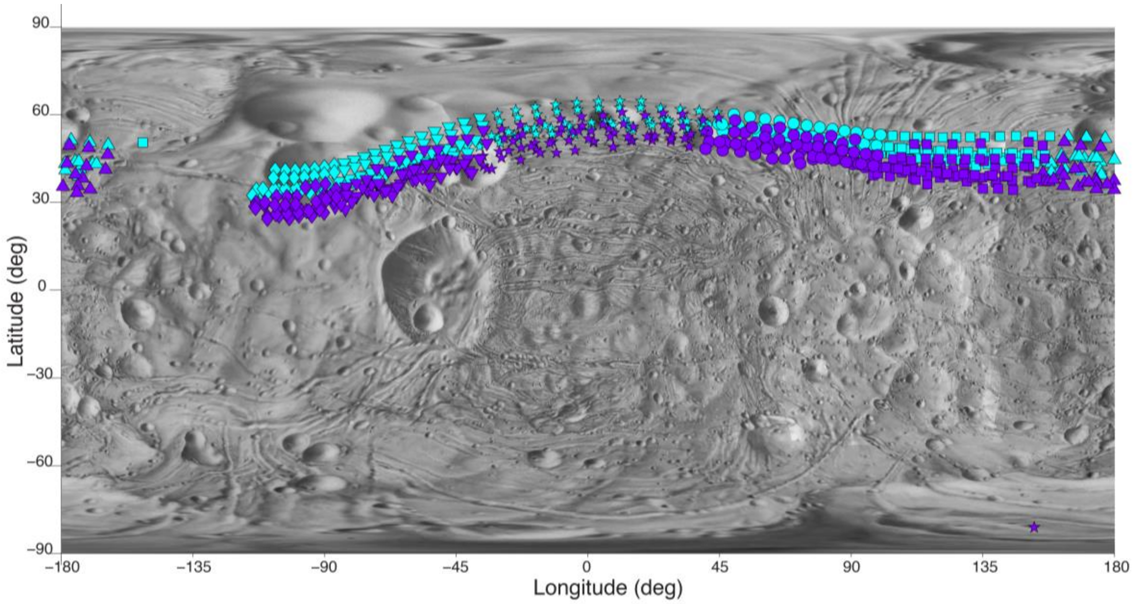

Supplementary Fig. 7. Variation of resultant catenae with Z-number (ejection angle). Reaccretion map for ejecta from the Grildrig crater, at Phobos periapsis around Mars, where the ejection angle is held fixed at 56.3° (blue catena) and allowed to vary stochastically between 45° and 65° (purple catena). Highly similar reaccretion patterns between the two methods show that the catena formation mechanism detailed here does not depend on *Z*-number or ejection angle variations.
